# The Levels of Circulating Proangiogenic Factors in Migraineurs

**DOI:** 10.1007/s12017-017-8465-7

**Published:** 2017-09-16

**Authors:** Slawomir Michalak, Alicja Kalinowska-Lyszczarz, Danuta Wegrzyn, Anna Thielemann, Krystyna Osztynowicz, Wojciech Kozubski

**Affiliations:** 1 Department of Neurochemistry and Neuropathology, Poznan University of Medical Sciences, 49, Przybyszewskiego Str., 60-355 Poznan, Poland; 2 Department of Laboratory Diagnostics, Poznan University of Medical Sciences, Poznan, Poland

**Keywords:** Migraine, Cerebrovascular disease, Vascular endothelial growth factor (VEGF), Angiogenin, Angiopoietin-2

## Abstract

Migraine has been reported as a risk factor for ischemic stroke or cardiovascular events, and dysfunction of endothelial cells has been evidenced in migraine patients. Proangiogenic factors are potential endothelial stimulators, and their disturbances can link abnormalities of endothelium with increased risk of vascular disorders. The aim of this study was to evaluate the levels of circulating proangiogenic factors in sera of migraineurs during interictal period. Fifty-two patients aged 37.9 ± 9.6 years, fulfilling International Headache Society criteria for migraine, were included in this observational case–control study. The control group included 39 healthy volunteers, matched according to age and gender. All subjects underwent full neurological examination and clinimetric evaluation with the use of: MIDAS, MIGSEV, QVM, VAS and VRS scales. Serum concentrations of vascular endothelial growth factor (VEGF), angiogenin, angiopoietin-2, thrombopoietin and Tie-2 were estimated in migraineurs and in the control group with the use of ELISA. In migraineurs during interictal period, we have found decreased serum VEGF and angiogenin concentrations compared with controls. Age of migraine onset correlated with VEGF, angiopoietin-2 and thrombopoietin concentrations. Furthermore, angiopoietin-2 level correlated with QVM score and Tie-2 with pain intensity evaluated using MIGSEV scale. In migraine patients during interictal period, depletion of VEGF and angiogenin, two cooperating proangiogenic factors, can be responsible for endothelial dysfunction and increased risk for vascular disorders.

## Introduction

The evidence of association between migraine and cardiovascular events, such as ischemic stroke, has long been reported. The results of The Stroke Prevention in Young Women Study showed increased risk of ischemic stroke among women with probable migraine with visual aura (MacClellan et al. 2007). In men with migraine, such increased risk of cardiovascular diseases is driven by increased risk of myocardial infarction (Kurth et al. 2007). The risk of ischemic stroke was the highest in migraineurs with no history of hypertension, diabetes or myocardial infarction, but with a history of cigarette smoking and increased sevenfold (MacClellan et al. 2007). Case-controlled studies showed association between migraine with aura and increased risk of ischemic stroke of early onset (Etminan et al. 2005).

The involvement of genetic factors in migraine and its linkage to cardiovascular disorders is supported by a number of studies, which are presented in brief in Table 1. Some genetic abnormalities associated with migraine lead to endothelial dysfunction, such as C677T MTHFR polymorphism (Pezzini et al. 2007).

**Table 1:** Genetic factors linking migraine and cardiovascular disorders

	Description of finding	Reference
*Notch3* mutation (EGF-like extracellular domain)	22% of patients with CADASIL, that is characterized by subcortical transient ischemic attacks or strokes, have migraine with aura Abnormalities in Notch3 protein folding, dimerization and interactions with its ligands may cause the loss of its protective role against apoptosis in vascular smooth muscle cells	Joutel et al. (1997)
Unknown	Association with autosomal dominant vascular retinopathy and Raynaud’s phenomenon in a Dutch family	Terwindt et al. (1998)
*C677T MTHFR* polymorphism	This polymorphism increases both the risk of migraine with aura and the risk of ischemic stroke Patients with migraine with aura have higher prevalence of the homozygous *TT* genotype and of the *T* allele of the polymorphism which leads to reduction of *MTHFR* activity Migraine and the *TT* genotype are strongly associated with the subgroup of patients with spontaneous cervical artery dissection, when compared to patients with non-cervical artery dissection ischemic stroke and controls *MTHFR* is involved in degradation of homocysteine which protects against homocysteine-related endothelial dysfunction that may further activate trigeminal fibers, induce inflammatory reaction, vasodilatation and finally migraine attack	Pezzini et al. (2007) Bellamy and McDowell (1997)
*NOS3* gene	Conflicting results: In one study, association with glaucoma and migraine was noted (Logan et al.), and in another one, no such linkage was observed (Griffiths et al.)	Logan et al. (2005) Griffiths et al. (1997)

*CADASIL* cerebral autosomal dominant arteriopathy with subcortical infarcts and leukoencephalopathy, *EGF*-*like* epidermal-growth-factor-like gene, *MTHFR* methylenetetrahydrofolate reductase, *NOS3* nitric oxide synthase

The study on the structure and function of cranial and peripheral arteries showed abnormalities in migraine patients (de Hoon et al. 2003). The temporal artery diameter was larger, and distension of brachial artery was smaller in migraineurs, when compared to controls. Brachial artery showed increased stiffness and intima-media thickness in migraine patients (de Hoon et al. 2003). What is more, flow-mediated dilatation of the brachial artery, which reflects endothelium-dependent vasodilatation capacity, is decreased in migraineurs (Vanmolkot et al. 2007).

Endothelial dysfunction may link genetic determinants and vascular abnormalities in migraine patients. Recent study (Lee et al. 2008) showed reduced number and decreased functions of circulating endothelial progenitor cells in migraine patients. The impairment of endothelial function leads to increased risk of cerebrovascular and cardiovascular diseases.

Increased frequency of anti-endothelial antibodies has been shown in migraine patients (Gabrielli et al. 2002). Thus, the pathomechanism behind the vascular complications of migraine should include genetics, endothelium function modulators and autoimmune factors. Endothelial function is modified by regulators of angiogenesis. The aim of the present study was to evaluate the levels of circulating proangiogenic factors in sera of migraineurs during interictal period. The rationale for the study was based on previous observations of endothelial dysfunction in the course of migraine and associated cardiovascular disorders.

## Patients and Methods

Fifty-two migraineurs, aged 37.9 ± 9.6 years, fulfilling International Headache Society criteria for migraine were included in the study. The study was designed as observational, case–control. Patients were recruited between 2005 and 2007 in the Department of Neurology, Poznan University of Medical Sciences, in the outpatient clinic. Written informed consent was obtained from all the participants. The study protocol was approved by the Internal Review Board at the Poznan University of Medical Sciences. To eliminate other factors which may influence vascular function, we excluded all subjects with history of cardiovascular disease, hypertension (defined as systolic blood pressure exceeding 140 mm Hg or diastolic blood pressure over 90 mm Hg), diabetes, hyperlipidemia, pregnancy or lactation, inflammation, allergy and regular use of vasoactive drugs (except hormonal contraceptives). Patients treated chronically with any drugs were also excluded from the study.

A full neurological examination was performed in all the subjects, including clinimetric evaluation with the use of: MIGSEV (El Hasnaoui et al. 2003), MIDAS (Stewart et al. 2001), QVM (Qualité de Vie et Migraine) (Richard et al. 1993), VAS (visual analog scale: 0–10 points, with 10 indicating the most severe pain) and VRS (four-point verbal rating scale: 0–3, with 3 for severe pain). Blood samples were collected not earlier than 4 days after migraine attack and/or administration of triptans or ergot alkaloids.

Based on medical history, physical examination and routine laboratory tests, none of the migraineurs or controls showed symptoms of any active or chronic disease. The control group consisted of 39 healthy volunteers aged 38.9 ± 7.0 years, matched to the study group according to age and gender.

Both migraineurs and controls were tested for laboratory markers of inflammation: white blood cells count (WBC) and high sensitivity C-reactive protein (hsCRP), IgE level, anti-dsDNA (anti-double stranded DNA), anti-MPO/pANCA (anti-myeloperoxidase/perinuclear pattern anti-neutrophil cytoplasm autoantibodies), anti-Pr3/c-ANCA (anti-proteinase 3/cytoplasmic pattern anti-neutrophil cytoplasm autoantibodies), ASMA (anti-smooth muscle antibodies), APCA (anti-parietal cell antibodies), AMA (antimitochondrial antibodies), HMA (hepatocyte membrane antibodies) and anti-cardiolipin antibodies (IgM and IgG). Total cholesterol, HDL-cholesterol, LDL-cholesterol, triglycerides, Lp(a) and homocysteine concentrations were also estimated in the sera of migraine patients and healthy subjects.

Serum concentrations of vascular endothelial growth factor (VEGF), angiogenin, angiopoietin-2, thrombopoietin and Tie-2 were estimated in migraineurs and control group with the use of ELISA (Quantikine, R&D).

Statistical analysis, using MedCalc Statistical Software version 15.8 (MedCalc Software bvba, Ostend, Belgium; https://www.medcalc.org; 2015), included the nonparametric Mann–Whitney *U* test for comparing two different groups. Summary statistics for continuous normally distributed variables was calculated as mean, standard deviation and range. For ordinal variables and non-normally distributed continuous variables median, first and third quartiles and range were calculated. Spearman rank correlation coefficients were calculated to assess the associations between different variables. The *p* value <0.05 was considered statistically significant.

## Results

Clinimetric data of migraine patients included in the study are presented in Table 2. Based on MIGSEV score, we classified our migraineurs cohort as Grade 2 (intermediate) to Grade 3 (high severity). MIDAS scale mean scoring (over 21 points), as well as Global Index on QVM scale, indicates severe disability in our patients. The mean scores from visual analog scale and VRS (see Table 2) also show high intensity of pain in migraineurs included in the study. Twenty-three percent of migraineurs (*n* = 12) were treated for attacks with drugs exhibiting a possible effect on circulating proangiogenic factors: Nine patients (17.3%) were treated with triptans, two (3.8%) with ergot alkaloids, and one patient (1.9%) was treated with both. The rest of migraineurs used non-steroid anti-inflammatory drugs during attacks.

**Table 2:** Clinimetric characteristics of migraineurs included in the study

	Migraine with aura *N* = 25	Migraine without aura *N* = 27	*p*
VAS (visual analog scale)	8 ± 1	8 ± 1	0.24
VRS (four-point verbal rating scale)	2.8 ± 0.4	2.9 ± 0.3	0.11
QVM *Global Index*	26.7 ± 6.1	27.1 ± 5.3	0.63
MIDAS	38 ± 18	41 ± 19	0.18
MIGSEV—*pain*	3.5 ± 0.5	3.5 ± 0.5	0.80
MIGSEV—*nausea*	3.3 ± 0.6	3.3 ± 0.5	0.71
MIGSEV—*disability in daily activity*	3.2 ± 0.7	3.2 ± 0.5	0.46
MIGSEV—*tolerability*	2.5 ± 0.7	2.6 ± 0,6	0.31

The markers of inflammation that we tested were not elevated in either migraineurs or the controls. White blood cells count did not differ between migraine patients (5.28 ± 1.48 × 10^9^/L) and controls (6.38 ± 1.59 × 10^9^/L). The concentration of hsCRP in migraine patients (median 0.42; interquartile range, IQR, 0.19–0.75 mg/dL) was similar to that of the control group (median 0.46; IQR 0.16–1.09 mg/dL). No differences were found in IgE levels between studied groups (median 32.4; IQR 20.2–59.3 IU/mL and median 29.2; IQR 20.2–55.8 IU/mL; migraineurs and controls, respectively).

To eliminate the effect of autoantibodies on the levels of proangiogenic factors, we have estimated the presence and the levels of anti-cardiolipin antibodies, which did not differ between migraine patients and the healthy subjects (Table 3).

**Table 3:** Risk factors for atherosclerosis and autoantibodies in migraineurs and controls

	Control group	Migraine patients	*p*
Total cholesterol (mg/dL)	147 ± 35	217 ± 42	<0.001
TAG (mg/dL)	84 ± 22	103 ± 46	0.025
HDL (mg/dL)	66 ± 21	67 ± 13	0.79
LDL (mg/dL)	100 ± 18	135 ± 37	<0.001
Homocysteine (mmol/L)	12.82 ± 3.98	13.2 ± 5.2	0.73
Lp (a) (g/L) median (interquartile range)	0.065 (0.03–0.10)	0.09 (0.04–0.21)	0.15
Anti–nuclear antibodies Anti–dsDNA Anti-MPO pANCA Anti-Pr3 c-ANCA ASMA APCA AMA HMA Anti-cardiolipin IgM (U/mL) Anti-cardiolipin IgG (U/mL)	Negative in all patients Negative in all patients Negative in all patients Negative in all patients Negative in all patients Negative in all patients Negative in all patients Negative in all patients 1.30 (0.80–1.60) 0.60 (0.30–1.40)	Negative in all subjects Negative in all subjects Negative in all subjects Negative in all subjects Negative in all patients Negative in all patients Negative in all patients Negative in all patients 1.10 (0.90–1.80) 0.40 (0.10–0.60)	

*Anti*-*dsDNA* anti-double stranded DNA, *anti*-*MPO/pANCA* anti-myeloperoxidase/perinuclear pattern anti-neutrophil cytoplasm autoantibodies, *Anti*-*Pr3/c*-*ANCA* anti-proteinase 3/cytoplasmic pattern anti-neutrophil cytoplasm autoantibodies, *ASMA* anti-smooth muscle antibodies, *APCA* anti-parietal cell antibodies, *AMA* antimitochondrial antibodies, *HMA* hepatocyte membrane antibodies

The results of atherosclerosis risk factors estimations are presented in Table 3. We have found increased concentrations of total cholesterol, triglycerides and LDL-cholesterol in migraine patients compared to the control group; however, the mean values of triglycerides and LDL-cholesterol concentrations did not exceed reference values; only the total cholesterol concentration was slightly higher than the upper reference limit. We have also calculated BMI in migraineurs which was within normal limits and reached the mean value of 23 ± 1 (±SD).

In migraineurs during interictal period, we have found decreased serum VEGF concentration compared to controls (*p* = 0.03), see Table 4. Similarly, angiogenin level was decreased (*p* = 0.02) in migraine patients, see Table 4. No significant differences were found between the levels of angiopoietin, Tie and TPO in migraine patients and healthy subjects. We have not found differences in the levels of proangiogenic factors between migraineurs with and without aura, see Table 5.

**Table 4:** The levels of circulating proangiogenic factors in migraine patients during the interictal period and in controls

	Controls	Migraineurs	*p*
VEGF [pg/mL] (median; interquartile range)	390 157.0–678.2	212 149.5–392.2	0.027
Angiopoietin [pg/mL] (median; interquartile range)	361 311–608	382 323–469	0.98
Tie-2 [ng/mL] (median; interquartile range)	1.0 0.00–3.00	2.0 0.0–3.0	0.81
Angiogenin [pg/mL] (median; interquartile range)	1747 1478.0–1858.5	1373.5 1183–1692	0.016
Thrombopoietin [pg/mL] (median; interquartile range)	12.0 0.00–31.50	0.0 0.00–15.0	0.19

**Table 5:** Proangiogenic factors in migraineurs with and without aura

	Migraine with aura	Migraine without aura	*p*
VEGF [pg/mL] [median (interquartile range)]	282 162–454	264 133–450	0.62
Angiopoietin [pg/mL] [median (interquartile range)]	746 339–493	419 361 562	0.35
Tie-2 [ng/mL] [median (interquartile range)]	1.97 1.8–2.3	1.97 1.7–2.2	0.86
Angiogenin [pg/mL] [median (interquartile range)]	1452 1143–1805	1425 1208–1797	0.83
Thrombopoietin [pg/mL] [median (interquartile range)]	8.7 3.1–16.3	12.3 4.9–28.8	0.32

*VEGF* vascular endothelial growth factor

We have also taken into consideration the abortive treatment during migraine attacks and its effects on the levels of circulating proangiogenic factors. No effect of triptans (sumatriptan or zolmitriptan) or ergot alkaloids administration on the concentrations of VEGF, angiopoietin, Tie-2 and angiogenin was observed compared to patients treated with NSAIDS or analgesics (see Table 6).

**Table 6:** The effect of abortive treatment on the levels of proangiogenic factors

	Abortive treatment: triptans or ergot alkaloids	Abortive treatment: NSAIDs or analgesics	*p*
VEGF (pg/mL) [median (interquartile range)]	344 206–657	264 156–480	0.28
Angiopoietin (pg/mL) [median (interquartile range)]	365 326–608	383 340–519	0.93
Tie-2 (ng/mL) [median (interquartile range)]	2.09 1.9–2.29	1.94 1.6–2.2	0.38
Angiogenin (pg/mL) [median (interquartile range)]	1719 1140–1859	1431 1199–1811	0.91
Thrombopoietin (pg/mL) [median (interquartile range)]	14.7 3.9–30.2	8.3 2.5–17.7	0.38

*VEGF* vascular endothelial growth factor

The concentrations of VEGF (rS = 0.39, *p* = 0.01), angiopoietin-2 (rS = 0.33, *p* = 0.04) and thrombopoietin (rS = 0.38, *p* = 0.01) correlated with the age of first ever migraine attack. Furthermore, angiopoietin-2 level correlated with Functional Index on QVM score (rS = −0.34, *p* = 0.03) and Tie-2 with pain intensity evaluated using MIGSEV scale (rS = 0.33, *p* = 0.04) and Medical Index on QVM score (rS = −0.35, *p* = 0.02).

## Discussion

Our study revealed abnormalities in the levels of circulating proangiogenic factors in migraine patients. Three groups of angiogenesis regulators are currently defined: VEGF family members that play essential roles in angiogenesis or lymphangiogenesis, angiopoietin family members that regulate vascular stability as agonists of the Tie-2 receptor, fibroblast growth factor (FGF) family members and thrombospondin family members that inhibit angiogenesis.

VEGF and angiogenin have been demonstrated as the most significantly changed in migraineurs. VEGF represents a family of glycoproteins involved in angiogenesis, vasculogenesis, enhancement of vascular permeability, cytoprotection (Ferrara et al. 2003) and may act as a proinflammatory cytokine (Reinders et al. 2003). VEGF maintains protective action on endothelial cells. It increases the expression of nitric oxide synthase in endothelial cells and stimulates the production of nitric oxide (Bussolati et al. 2001). VEGF cytoprotective effect on endothelial cells results from induction of antiapoptotic genes, *Bcl*-*2* and *A1* (Gerber et al. 1998), stimulation of phosphatidylinositol 3′-kinase (PI3-kinase) activity and protein kinase C (Mason et al. 2004). There are lines of evidence for protective effect of VEGF against complement-mediated endothelial injury and of limiting leukocyte–endothelium interactions (Scalia et al. 1999).

The decrease in circulating VEGF during interictal period in migraine patients is therefore an important factor in a chain of pathomechanisms leading to endothelial dysfunction and increased risk of cardiovascular disorders.

In our study, we have carefully eliminated the effect of drugs used as abortive treatment during migraine attack. Since it has been shown that triptans (de Hoon et al. 2000) and ergot alkaloids (de Hoon et al. 2001) administration causes transient increase in arterial stiffness, we have collected the blood samples no earlier than 4 days after the attack. The effect of drugs on vascular properties is, however, short-lasting. Therefore, the period of 3 days between drug administration and blood sampling is sufficient even for the action of ergot alkaloids (de Hoon et al. 2001).

The effects of triptans on endothelial function are not clearly defined. On the one hand, the vasoconstrictive effect of 5-HT1B/1D receptor agonists is explained as a result of action on smooth muscles and insufficient release of vasodilatatory factors by endothelium (Golino et al. 1991). On the other hand, based on the observation of triptans action on endothelial function of brachial artery, its impairment after 5-HT1B/1D receptor agonists administration cannot be eliminated (de Hoon et al. 2000). Therefore, we excluded from the study any patients who used abortive treatment within 3 days before blood sampling.

Human angiogenin is a non-glycosylated polypeptide representing the RISBASE family of ribonucleases (Bond et al. 1993). It is expressed among others in vascular endothelial cells and in smooth muscle cells (Moenner et al. 1994), and is associated with angiogenesis. In the very early phase of this process angiogenin binds to actin, further the actin-ANG complex dissociates and subsequently tissue plasminogen activator is activated. Plasmin generation leads to degradation of basement membrane laminin and fibronectin (Hu and Riordan 1993). Destruction of the basement membrane is considered a prerequisite for endothelial cell migration during neovascularization (Hu et al. 1994). Angiogenin is a factor required for the action of other angiogenesis regulators, including VEGF (Kishimoto et al. 2005). This close cooperation between angiogenin and VEGF emphasizes our observation of lowered levels of both proangiogenic factors in migraine patients. Thus, low angiogenin concentration may aggravate the effects of decreased VEGF on endothelial dysfunction in our patients.

We have not found any changes in the levels of two other closely related proangiogenic factors, namely angiopoietin and Tie-2 receptor in migraine patients.

Angiopoietin-2 is a glycoprotein that regulates angiogenesis and is a ligand for the endothelial cell receptor tyrosine kinase Tie-2. Angiopoietin-2 mediates interactions between endothelial and perivascular cells and enhances the effects of proangiogenic proteins including VEGF (Maisonpierre et al. 1997). When acting alone, angiopoietin causes endothelial cell death; however, in concert with VEGF it leads to stimulation of angiogenesis (Lobov et al. 2002). The data on cooperation between angiopoietin and VEGF are significant for the interpretation of our study results. Despite unchanged levels of angiopoietin in migraine patients, depletion of VEGF may lead to endothelial damage as a result of angiopoietin action. The same suggestion can be made for Tie-2, which is a tyrosine kinase receptor expressed on endothelial cells, with angiopoietin as its ligand (Davis et al. 1996). Angiopoietin-2, whose levels were evaluated in this study, has the most important activity in the regulation of angiogenesis via interaction with Tie-2 receptor (Teichert-Kuliszewska et al. 2001).

We have included thrombopoietin in the group of evaluated proangiogenic factors, because, on the one hand, it stimulates angiogenesis (Brizzi et al. 1999), and on the other hand, it acts as a late-acting growth factor that exerts effects on the megakaryocyte, erythroid and granulocytic population (Wendling et al. 1994). The primary function of thrombopoietin is the regulation of platelets production; however, it was later shown to stimulate endothelial cells and VEGF cooperates with this function (Kanayasu-Toyoda et al. 2007). However, in our study the role of thrombopoietin in endothelial dysfunction seems rather neglectable.

The correlations of VEGF, angiopoietin-2 and thrombopoietin with the age of first ever migraine attack suggest that depletion of proangiogenic factors progresses with the duration of migraine. It is noteworthy that the results of clinimetric scoring correlated with concentrations of proangiogenic factors, such as angiopoietin and Tie-2, whose levels did not differ between migraineurs and controls. This may suggest a potential role of angiopoietin and Tie-2 in migraine pathophysiology.

Our study demonstrated increased total cholesterol, LDL-cholesterol and triglycerides concentrations in migraine patients, compared to controls. However, these observations have no significance for the explanation of VEGF and angiogenin depletion in migraineurs. It has previously been shown that hipercholesterolemia causes increase in VEGF concentration (Blann et al. 2001). The decreased angiogenin concentration was found in type 2 diabetes patients with hypertriglyceridemia, lower LDL-cholesterol and with HDL-cholesterol not significantly different from controls (Siebert et al. 2007). Such metabolic abnormalities cannot, however, explain abnormalities observed in migraine patients.

The possible limitation of our study is the sample size. The correlations that we have found are statistically significant (*p* < 0.05); however, they did not reach high significance of *p* < 0.001. Verification on a larger sample would be helpful in establishing the significance of our results.

With regard to future directions, it would be of interest to support our findings with measuring markers of endothelial dysfunction, such as adhesion molecules (i.e., intracellular adhesion molecule 1, ICAM-1), cytokines (interleukin 6, IL-6), matrix metalloproteinases. Also, in order to confirm that lower VEGF leads to endothelial dysfunction, one could correlate biochemical results with neuroimaging of brain vasculature, such as cerebral blood flow studies with the use of magnetic resonance imaging or positron emission tomography.

In conclusion, depletion of VEGF and angiogenin, two closely related proangiogenic regulators, in migraine patients during interictal period creates a milieu of factors that can be responsible for endothelial dysfunction and increased risk of vascular disorders.
